# Supplementary material for: MetaRibo-Seq measures translation in microbiomes
Source: Nat Commun. 2020 Jun 29;11:3268. doi: 10.1038/s41467-020-17081-z (PMC7324362; doi:10.1038/s41467-020-17081-z)
Supplement: Supplementary file 10 — Supplementary Data 7 [file 41467_2020_17081_MOESM10_ESM.zip › File2/Confidence_High_Taxonomy/84862_out.krona.html]

Javascript must be enabled to view this page.

members
magnitude
magnitudeUnassigned
count
unassigned
taxon
rank

84862\_out

6

4
superkingdom
2

4
phylum
1224

68525
subphylum
4

29547
4
class

213849
4
order

72293
family
4

4
genus
209

1548018
4

SRS013687\_contig\_number\_contig-100\_252.171833SRS023914\_contig\_number\_contig-100\_1609.161955SRS051031\_contig\_number\_contig-100\_1666.200622SRS1041145\_contig\_number\_40799
species

2759
2
superkingdom

kingdom
1
4751

1
subkingdom
451864

1
phylum
4890

subphylum
1
147538

class
1
147545

1
subclass
451871

1
order
5042

1131492
family
1

5052
1

SRS076929\_contig\_number\_contig-100\_1734.153737
genus

1
kingdom
33208

phylum
1
7711

89593
subphylum
1

8287
1
superclass

class
1
40674

314146
1
superorder

9443
1
order

1
suborder
376913

314293
infraorder
1

9526
parvorder
1

1
superfamily
314295

9604
family
1

1
subfamily
207598

9605
genus
1


SRS016095\_contig\_number\_1554
1
species
9606
